# Dynamic changes in HMGB1 levels correlate with inflammatory responses during cardiopulmonary bypass

**DOI:** 10.3892/etm.2013.1026

**Published:** 2013-03-22

**Authors:** ZHIWEI ZHANG, YUAN WU, YUAN ZHAO, XIANZHONG XIAO, JUNWEN LIU, XINMIN ZHOU

**Affiliations:** 1 Department of Cardiothoracic Surgery, The Second Xiang-Ya Hospital, Central South University, Changsha, Hunan 410011;; 2 Department of Pathophysiology, School of Basic Medical Sciences, Central South University, Changsha, Hunan 410078, P.R. China

**Keywords:** high mobility group box 1, cardiopulmonary bypass, tumor necrosis factor-α, systemic inflammatory response syndrome

## Abstract

High mobility group box 1 (HMGB1), which is released by activated immune cells and necrotic cells, has properties similar to those of pro-inflammatory cytokines. Cardiopulmonary bypass (CPB) induces systemic inflammation and aortic cross-clamping induces myocardial ischemia. This study was conducted to observe the dynamic changes of HMGB1 and tumor necrosis factor (TNF)-α levels during CPB and to analyze their clinical significance. A total of 78 cases of American Society of Anesthesiologists (ASA) grade II–IV undergoing elective valve replacement under CPB were included in this study. Blood and urine samples were collected after anesthesia prior to surgery (T1), before aortic cross-clamping (T2), after CPB (T3) and on the first day after surgery (T4), as well as the second (T5) and third (T6) day after surgery for determination of the levels of HMGB1, TNF-α, alanine aminotransferase (ALT), creatinine (Cr), blood urea nitrogen (BUN), N-acetyl-β-D-glucosamidase (NAG) and β2-microglobulin (β2-MG). Results revealed that: i) the serum levels of HMGB1 elevated as early as T1, increased until reaching a peak at T3, then decreased to a lower level at T4; ii) the serum level of TNF-α was low at T1, gradually increased in a similar manner to HMGB1, then decreased following CPB and reached the lowest point at T5; and iii) the levels of HMGB1 were positively correlated with serum TNF-α and serum ALT at T3. In conclusion, HMGB1 levels may be used as an indicator of inflammation and may be a novel target for controlling inflammation during CPB. The optimal treatment time is T3 (after CPB).

## Introduction

Cardiopulmonary bypass (CPB) is a technique that temporarily takes over the function of the heart and lungs during surgery, maintaining the circulation of blood and oxygen supply for the body. This technique is widely used during heart surgery due to the difficulty of operating on a beating heart. Surgery opens the chambers of the heart, which requires the use of CPB to support the circulation. However, since the body is under an abnormal status during CPB, an inflammatory response may be induced by multiple stimulants, including ischemia-perfusion injury, low temperature and CPB time. This is likely to result in the occurrence of multiple organ dysfunction syndrome (MODS) (1,2). Therefore, studies on the systemic inflammatory response during CPB are crucial for the prevention and treatment of complications following CPB. Additionally, pro-inflammatory cytokines not only reflect the condition of the systemic inflammatory response, but are also involved in the occurrence of complications (3).

High mobility group box-1 (HMGB1) is a multifunctional protein, described as a non-histone DNA-binding nuclear protein. HMGB1 binds to DNA in a sequence-independent manner and modifies DNA structure to facilitate transcription, replication and repair (4). This function is essential for survival, as HMGB1-deficient mice die of hypoglycemia within 24 h of birth (5). Previously, HMGB1 was identified as a cytokine mediator for lethal systemic inflammation, arthritis and local inflammation (6,7). In a previous study, it was observed that the addition of purified recombinant HMG-1 to human monocyte cultures significantly stimulated the release of tumor necrosis factor (TNF), which is an important pro-inflammatory mediator. The HMG-1-activated TNF synthesis occurred in a biphasic pattern, with an early peak at 3 h after the addition of HMG-1, followed by another peak at 8-10 h (8). However, the LPS-induced TNF release only peaked 1 h after LPS administration. As for the changes in TNF level during CPB, Hennein *et al* identified that arterial TNF-α increases in a bimodal manner, peaking at 2 and 18–24 h after surgery, then declines to the level before bypass (9).

However, the expression of HMGB1 during CPB remains unclear. Therefore, in the present study, the levels of HMGB1 expression before, during and after surgery were characterized, as well as the levels of TNF-α. Given the close association between HMGB1 and TNF-α, the correlation between HMGB1 and TNF-α was analyzed. Exploring the role of HMGB1 in inflammation may provide information beneficial in further intervention to reduce the related inflammatory response.

## Patients and methods

### Patients

Seventy-eight patients (41 males and 37 females) aged 24–59 years with an American Society of Anesthesiologists (ASA) grade of II–IV for heart function and rheumatic heart disease requiring valve replacement surgery were admitted to the Department of Cardiothoracic Surgery, Second Xiangya Hospital, Central South University between May 1, 2007 and November 31, 2008. Informed consent was obtained from patients or close relatives of patients. The study was conducted in accordance with the Declaration of Helsinki and was approved by the research ethics committee at the Central South University.

The patients had no medical history of endocarditis, diabetes, hypertension, neurological diseases, immune diseases or psychiatric diseases, as well as infectious diseases, including tuberculosis, hepatitis B and syphilis. The function of the liver, kidneys and lungs was normal prior to surgery. There were three types of surgery, including mitral valve replacement (MVR, n=40), aortic valve replacement (AVR, n=14) and double valve replacement (DVR, n=24).

### Collection of blood samples

The CPB was performed as previously described (10). Blood or urine samples were collected after anesthesia prior to surgery (T1), before aortic cross-clamping (T2), after CPB (completion of cross-clamping; T3) and the first (T4), second (T5) and third (T6) day after surgery between 6 and 7 a.m. Levels of HMGB1, TNF-α, serum alanine aminotransferase (ALT), serum creatinine (Cr), blood urea nitrogen (BUN), urinary N-acetyl-β-D-glucosamidase (NAG), urinary Cr and urinary β2-microglobulin (β2-MG) were determined. In order to exclude the effect of blood dilution during CPB on the detection values, the final results were corrected as follows: Corrected value = actual value x hematocrit (HCT) before aortic cross-clamping/HCT when clamping.

### Measurement of cytokines

Serum TNF-α was quantified using an enzyme-linked immunosorbent assay (ELISA) kit (Boster Biological Technology Ltd., Wuhan, China) and expressed in picograms per milliliter. Absorbance was determined at 450 nm using a microplate reader (BioTek Instruments Inc., Winooski, VT, USA).

### Western blot assay

Following routine laboratory tests, an equal volume of blood serum was collected and filtered through Millex-GP (Millipore, Bedford, MA, USA) to remove debris and other macromolecular complexes. Samples were then concentrated 40-fold with Amicon Ultra-4-10000 NMWL (Millipore) following the manufacturer’s instructions, as previously described (11,12). Proteins were then resolved by 10% sodium dodecyl sulfate-polyacrylamide gel electrophoresis (SDS-PAGE) and transferred onto polyvinylidene difluoride (PVDF) membranes (Schleicher & Schuell Bioscience Inc., Keene, NH, USA). Membranes were blocked with 2% bovine serum albumin for 6 h and incubated for 2 h with a primary antibody specific for HMGB1 (1:1000; 556528; BD Biosciences, San Jose, CA, USA). Following incubation with peroxidase-conjugated secondary antibody for 1 h at room temperature (RT), the bands were visualized by diaminobenzidine detection (Boster Biological Technology Ltd.) according to the manufacturer’s instructions and semi-quantitatively determined using Gel-Pro Analyzer densitometry software (Media Cybernetics, Bethesda, MD, USA).

### Statistical analysis

All descriptive data are expressed as mean ± standard error of the mean (SEM) for continuous variables. Comparisons of two groups were performed by student’s t-test. Multiple comparisons were performed by one- or two-way analysis of variance (ANOVA) followed by Bonferroni’s test. The Pearson correlation coefficient test was used to evaluate associations between two quantitative variables. P<0.05 was considered to indicate a statistically significant difference.

## Results

### Changes to HMGB1 expression level during CPB

**Figure 1:**
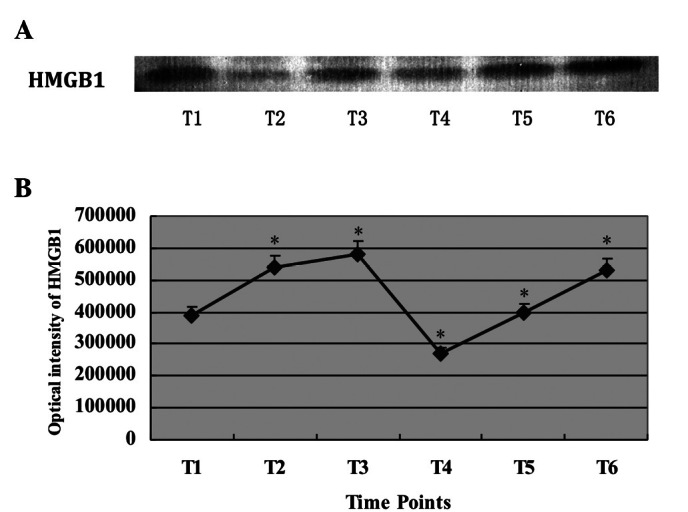
Expression level of HMGB1 during CPB. (A) Representative bands of HMGB1 protein expression at different time points. (B) Corrected relative optical intensity values of HMGB1 at different time points. T1, before surgery (after anesthetization); T2, before aortic cross-clamp; T3, after CPB; T4, first day after surgery (between 6 and 7 a.m.); T5, second day after surgery (between 6 and 7 a.m.); T6, third day after surgery (between 6 and 7 a.m.). ^*^P<0.05, vs. T1. Data are expressed as mean ± SEM, n=78. HMGB1, high mobility group box 1; CPB, cardiopulmonary bypass; SEM, standard error of the mean.

The results of western blot analysis revealed that the protein level of HMGB1 at T3 was much higher than at T1 and T2. The level at T4 was significantly lower than at T3 (P<0.01). Subsequently, the protein levels of HMGB1 were increased at T4, T5 and T6 (Fig. 1A).

The relative optical intensity values of the results were corrected based on the HCT using the formula described in Materials and methods. The values at T2 and T3 were not corrected due to the CPB. Summarized data in Fig. 1B revealed that HMGB1 was expressed as early as T1 (before surgery, after anesthesia), increased after T2 (cross-clamping) and peaked at T3 (completion of cross-clamping). After surgery (T4), the HMGB1 level was rapidly decreased to the level measured at T1; however, it increased again at T5 and further increased on the third day after surgery (T6), at which time it was close to the level at T2.

### Changes to TNF-α level during CPB

**Figure 2:**
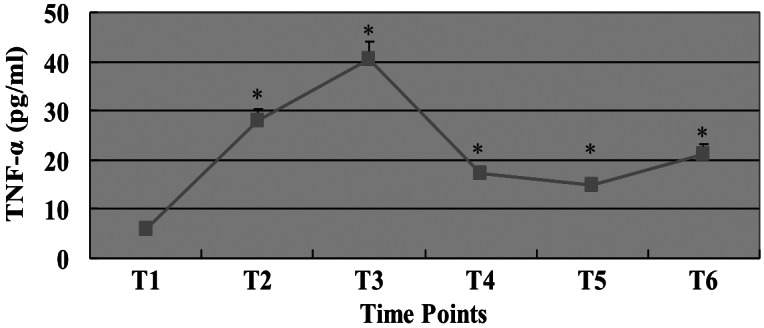
Serum level of TNF-α during CPB. The serum level of TNF-α at each time point is shown. T1, before surgery (after anesthetization); T2, before aortic cross-clamping; T3, after CPB; T4, first day after surgery (between 6 and 7 a.m.); T5, second day after surgery (between 6 and 7 a.m.); T6, third day after surgery (between 6 and 7 a.m.). ^*^P<0.05, vs. T1. Data are expressed as mean ± SEM, n=78. TNF, tumor necrosis factor; CPB, cardiopulmonary bypass; SEM, standard error of the mean.

As shown in Fig. 2, the level of TNF-α increased from the beginning of CPB and peaked at the end of cross-clamping (T3). The level of TNF-α decreased significantly after surgery (T4), and the reduction lasted to the second day after surgery; however, the TNF-α levels remained higher than the level recorded before surgery (T1). On the third day after surgery (T6), the level of TNF-α was increased slightly; however, it remained lower than the level at T2.

**Figure 3:**
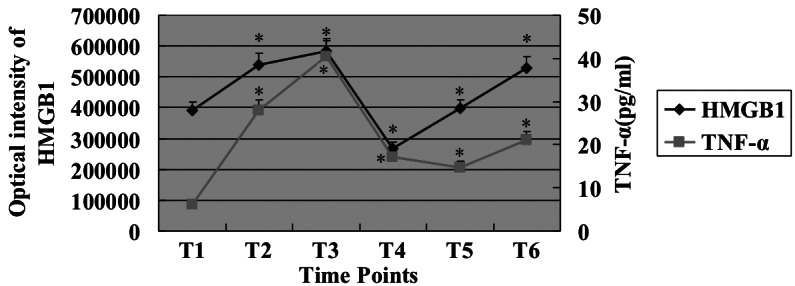
Merged curve of HMGB1 and TNF-α levels. The overlapped figure shows that profiles of HMGB1 and TNF-α are similar. T1, before surgery (after anesthetization); T2, before aortic cross-clamping; T3, after CPB; T4, first day after surgery (between 6 and 7 a.m.); T5, second day after surgery (between 6 and 7 a.m.); T6, third day after surgery (between 6 and 7 a.m.). ^*^P<0.05, vs. T1. Data are expressed as mean ± SEM, n=78. HMGB1, high mobility group box 1; TNF, tumor necrosis factor; CPB, cardiopulmonary bypass; SEM, standard error of the mean.

The merged curves of HMGB1 and TNF-α are shown in Fig. 3. The curve profile of HMGB1 level was similar to the curve of TNF-α level.

### Changes to other parameters during CPB

The clinical parameters were determined and presented in Table I, together with the levels of HMGB1 and TNF-α. As shown in Table I, changes of white blood cell (WBC) levels, neutrophils (N), lymphocytes (L), BUN, Cr and ALT during CBP were detected and the results were consistent with those of a previous study (13). No significant changes of kidney function were observed following surgery. ALT increased following CPB and reached a peak at T6 without any decrease. Changes in liver function following surgery appeared to be correlated with the expression of HMGB1. Therefore, a correlation analysis between these parameters was performed.

### Correlations between HMGB1, TNF-α and other parameters

HMGB1 was positively correlated with TNF-α (r= 0.237, P= 0.002). ALT was positively correlated with HMGB1 (r=0.559, P=0.02) but not with TNF-α (r=-0.49; P=0.858) at T3. The correlations between NAG/Cr and HMGB1 (r=0.376; P=0.379) or TNF-α were negative (r=0.236; P=0.134) at T3.

## Discussion

In this study, we demonstrated the significant changes in serum levels of HMGB1 and TNF-α following CPB, as well as other parameters. This is the first study to track the changes of HMGB1 level during CPB at six time points: before surgery, before aortic cross-clamping, after CPB and one, two and three days after surgery. The HMGB1 levels were significantly different in the patients with CPB and may be correlated with the level of TNF-α.

It is well known that CPB is characteristically associated with a systemic inflammatory response, which is initiated by the synthesis of various cytokines, including TNF, inter-leukin-1, interleukin-6 and interleukin-8 (14). Inflammatory cytokines, endothelial activation and endothelial-leukocyte interactions have been reported to play an important role in the induction of systemic inflammatory response syndrome (SIRS), causing large fluid shifts, coagulation disturbances and increased concentrations of catecholamines and stress hormones (15). Several reports have shown that marked changes in serum cytokine levels occur during and after CPB in children and adults (16,17). An increased inflammatory response due to elevated sequestration of inflammatory mediators is considered responsible for the high incidence of poor outcomes following bypass (16).

HMGB1 was first identified as a nuclear protein; then, it was revealed that HMGB1 activates inflammatory responses through multiple pathways, including macrophage activation and release of pro-inflammatory cytokines, endothelial cell activation and expression of adhesion molecules. These effects of HMGB1 lead to a cascade of inflammatory responses that cause tissue damage and even mortality (11). HMGB1 is secreted as a late mediator during inflammation and participates in the pathogenesis of systemic inflammation once the early mediator response has resolved. *In vitro*, extracellular HMGB1 activates macrophages and monocytes and promotes dendritic cell maturation (18,19). *In vivo*, HMGB1 causes acute lung inflammation, epithelial-cell barrier leakage and even mortality (20). Extracellular HMGB1 translocation during inflammatory responses *in vivo* leads to significantly increased serum levels in patients with arthritis, sepsis and other inflammatory disorders (21). Moreover, increased levels of HMGB1 are observed in patients with sepsis and other major inflammatory diseases, including rheumatoid arthritis (22). In the current study, we identified that HMGB1 is expressed initially before surgery after anesthetization (T1). Then, it increases following cross-clamping (T2) and peaks at the completion of cross-clamping (T3). A factor involved in the increase of HMGB1 may be stress. Additionally, this change may result from myocardial ischemia/reperfusion during cardiac surgery. Following surgery, the HMGB1 level rapidly decreased to the level before surgery (T4) and then increased again (T5), and the elevation continued until the third day after surgery (T6). The level of HMGB1 was high prior to surgery, possibly as a result of the long-term chronic inflammatory stimulation of rheumatic heart disease.

The cytokines that are significantly increased following bypass are also associated with SIRS/MODS. TNF-α is strongly associated with SIRS/MODS (23). Hypotension, followed by coagulopathy and renal dysfunction, is strongly associated with the increase of TNF-α. TNF-α may be a direct inducer of hypotension, coagulopathy and renal dysfunction (24–27). It was suggested that during bypass, an elevation of TNF-α may participate in capillary leakage and subsequently pulmonary edema, which may lead to respiratory dysfunction (28). In the present study, the level of TNF-α was monitored during CPB. Our data revealed that the TNF-α level was highest at T3 (after CPB) and the highest inflammatory reaction was observed at T3. These data suggest that anti-inflammatory treatment may be most beneficial after CPB. Moreover, we identified that the variation profile of TNF-α level with time was consistent with that of HMGB1, suggesting that HMGB1 and TNF-α are indicators of an inflammatory response. Due to the important role of TNF-α in pathophysiological processes during CPB, intervention targeting these two mediators may be effective. For example, potent inhibitors of TNF-α or HMGB1 prior to bypass may be useful in ameliorating the damage resulting from inflammatory responses.

In addition to the correlation between HMGB1 and TNF-α, we also investigated the correlations of HMGB1 and TNF-α with other important biochemical parameters during CPB. We identified that ALT was positively correlated with HMGB1, which may suggest that HMGB1 plays a role in hepatic injury during CPB. The mechanism may be the ischemia-reperfusion of multiple organs during CPB. This further supports a previous study suggesting that HMGB1 mediates hepatic injury following murine liver ischemiareperfusion (29).

The results of the present study demonstrate a positive correlation between HMGB1 and TNF-α, suggesting that HMGB1 is an indicator of inflammation during CPB. These data provide useful evidence to support further *in vivo* and *in vitro* studies aimed at therapy targeting HMGB1 to attenuate inflammation-related damage following surgery. In addition, considering the time-curve of TNF-α level during CPB, the optimal timing for treatment is after CPB at the completion of cross-clamping (T3).

## Figures and Tables

**Table I:** Clinical characteristics of patients during CPB.

Parameters	T1	T2	T3	T4	T5	T6
WBC (×10^9^ cells/l)	5.56±1.50	5.59±1.56	13.05±7.69^a^	18.74±6.62^a^	29.29±9.07^a^	22.96±7.91^a^
N (%)	61.78±14.30	58.75±10.47	75.22±13.57^a^	89.71±3.50^a^	87.89±3.40^a^	81.72±5.88^a^
L (%)	24.93±5.93	31.95±7.58^a^	22.61±12.58	5.73±2.82^a^	5.95±2.46^a^	10.99±5.25^a^
BUN (mmol/l)	6.25±2.61	8.36±2.16^b^	9.36±2.59^a^	11.02±3.64^a^	12.53±6.38^a^	10.98±6.81^a^
Serum Cr (*μ*mol/l)	63.87±13.43	94.07±24.17^a^	103.42±30.24^a^	111.45±38.63^a^	91.52±41.07^a^	85.73±31.18^b^
ALT (U/l)	23.43±10.17	19.67±9.10	22.38±8.22	32.24±8.50^a^	34.85±9.65^a^	43.67±25.87^a^
Urinary NAG/Cr (u/g.cr)	0.019±0.019	0.043±0.070^a^	0. 62±0.610^a^	0.013±0.006	0.045±0.030^a^	0.044±0.026^a^
Urinary β2-MG/Cr (mg/mmol)	0.01±0.01	0.93±0.26^b^	0.51±0.64^a^	0.20±0.29^a^	0.13±0.15	0.08±0.08
TNF-α (pg/ml)	6.01±5.51	27.96±17.80^a^	40.37±25.41^b^	17.18±12.26^a^	14.81±11.92^a^	21.19±19.52^a^
HMGB1 (pixels)	390228±205.5	539322±212.5^a^	583262±388.5^a^	268621±136.6^a^	397748±196.9	529287±278.5^a^

Data are presented as mean ± standard error of the mean. CPB, cardiopulmonary bypass; T1, before surgery, after anesthetization; T2, before aortic cross-clamp; T3, after CPB; T4, first day after surgery (between 6 and 7 a.m.); T5, second day after surgery (between 6 and 7 a.m.); T6, third day after surgery (between 6 and 7 a.m.); WBC, white blood cell; N, neutrophils; L, lymphocytes; BUN, blood urea nitrogen; Cr, creatinine; ALT, alanine aminotransferase; NAG, N-acetyl-β-D-glucosamidase; β2-MG, β2-microglobulin; TNF-α, tumor necrosis factor α; HMGB1, high mobility group box 1.

a P<0.01,

b P<0.05, vs. value at T1.
